# An evaluation of seasonal variations in footwear worn by adults with inflammatory arthritis: a cross-sectional observational study using a web-based survey

**DOI:** 10.1186/s13047-014-0036-7

**Published:** 2014-08-13

**Authors:** Angela Brenton-Rule, Gordon J Hendry, Georgina Barr, Keith Rome

**Affiliations:** 1 Division of Rehabilitation & Occupation Studies, AUT University, Private Bag 92006, Auckland 1020, New Zealand; 2 School of Health and Life Sciences, Glasgow Caledonian University, Cowcaddens Rd, Glasgow G4 0BA, Lanarkshire, UK

**Keywords:** Footwear, Pain, Adherence, Comfort, Seasonal variation, Inflammatory arthritis

## Abstract

**Background:**

Foot problems are common in adults with inflammatory arthritis and therapeutic footwear can be effective in managing arthritic foot problems. Accessing appropriate footwear has been identified as a major barrier, resulting in poor adherence to treatment plans involving footwear. Indeed, previous New Zealand based studies found that many people with rheumatoid arthritis and gout wore inappropriate footwear. However, these studies were conducted in a single teaching hospital during the New Zealand summer therefore the findings may not be representative of footwear styles worn elsewhere in New Zealand, or reflect the potential influence of seasonal climate changes. The aim of the study was to evaluate seasonal variations in footwear habits of people with inflammatory arthritic conditions in New Zealand.

**Methods:**

A cross-sectional study design using a web-based survey. The survey questions were designed to elicit demographic and clinical information, features of importance when choosing footwear and seasonal footwear habits, including questions related to the provision of therapeutic footwear/orthoses and footwear experiences.

**Results:**

One-hundred and ninety-seven participants responded who were predominantly women of European descent, aged between 46–65 years old, from the North Island of New Zealand. The majority of participants identified with having either rheumatoid arthritis (35%) and/or osteoarthritis (57%) and 68% reported established disease (>5 years duration). 18% of participants had been issued with therapeutic footwear. Walking and athletic shoes were the most frequently reported footwear type worn regardless of the time of year. In the summer, 42% reported wearing sandals most often. Comfort, fit and support were reported most frequently as the footwear features of greatest importance. Many participants reported difficulties with footwear (63%), getting hot feet in the summer (63%) and the need for a sandal which could accommodate a supportive insole (73%).

**Conclusions:**

Athletic and walking shoes were the most popular style of footwear reported regardless of seasonal variation. During the summer season people with inflammatory arthritis may wear sandals more frequently in order to accommodate disease-related foot deformity. Healthcare professionals and researchers should consider seasonal variation when recommending appropriate footwear, or conducting footwear studies in people with inflammatory arthritis, to reduce non-adherence to prescribed footwear.

## Background

Inflammatory arthritis affects more than 500,000 New Zealanders [1]. Foot problems are commonly associated with adult arthritic conditions, particularly with rheumatoid arthritis (RA) [2],[3] and other inflammatory arthritic conditions such as gout [4], systemic sclerosis [5], psoriatic arthropathy [6], systemic lupus erythematous [7] and osteoarthritis (OA) [8]. Progressive foot problems affect up to 90% of patients with established RA [2],[3] and up to 70% of initial gout flares occur in the first metatarsophalangeal joint of the foot [9]. Patients with inflammatory arthritis have complex needs and foot problems can be inadequately understood or overlooked during consultations [3] despite previous studies which suggest that non-pharmacological interventions can reduce pain and disability and improve long-term outcomes for patients with existing and potential foot problems [10]––[12].

Podiatrists are often integrated members of multidisciplinary rheumatology teams and deliver foot care comprised of palliative skin and nail care, wound care, orthotic management, and therapeutic footwear [13],[14]. Therapeutic footwear, whether it is off-the-shelf, custom-made or retail, has been shown to be effective in managing foot problems associated with RA [11],[15],[16], and may reduce foot pain and musculoskeletal disability in people with gout [10]. A previous study has shown that for people with RA, comfort and fit are reported as the major factors that influence footwear choice [17]. Further, patients with gout report that comfort, fit, provision of support, and cost are the main influencing factors over their footwear choices [18]. However, difficulties can develop where structural foot changes attributable to the arthritis progression make it problematic for patients to find appropriate shoes that are capable of accommodating their foot deformities. Martini et al. [19] reported that people with gout, who experienced symptoms in their lower extremities, were unable to put their shoes on or walk with ease and subsequently stayed at home. To get around the home, they limped, used crutches, or relied on family support to be mobile [19]. For people with RA-related foot problems, footwear difficulties can be the source of considerable distress and reinforce negative feelings associated with arthritic foot changes [15].

Appropriate footwear for people with inflammatory arthritis has been identified as a major barrier relating to adherence to treatment plans involving prescribed footwear [15],[20],[21]. Previous studies looking at footwear characteristics associated with RA [17] and gout [18] in New Zealand, also found that many people wore inappropriate footwear. In the case of gout, this was in part due to the financial limitations of those affected by the illness [18]. However, these studies lacked external validity as patients were recruited from one large teaching hospital during the summer months only. Therefore, these findings may not be a true representation of footwear styles worn elsewhere in New Zealand, nor do they capture the potential influence of seasonal climate changes. A qualitative study of people with RA in Australia found that higher temperatures experienced during summer may play a key role in influencing footwear habits [20]. Moreover, further qualitative research conducted in the UK identified the frustration amongst women with RA due to their inability to follow seasonal footwear trends [22]. The current research consists of a nationwide survey which aims to identify the features of importance when choosing footwear and the type of footwear worn by people with inflammatory arthritis during the winter and summer months. The aim was to survey footwear habits of people with inflammatory arthritic conditions in New Zealand and to identify any seasonal variation.

## Methods

A cross-sectional observational study design using a web-based survey was used. Participants were a convenience sample of adults who were identified as having inflammatory arthritis. The survey was promoted by Arthritis New Zealand. Currently, over 4000 members of Arthritis New Zealand have been registered as having inflammatory joint disease including RA, gout, psoriatic arthritis, osteoarthritis, systemic lupus erythematous, juvenile idiopathic arthritis, fibromyalgia, systemic sclerosis/scleroderma and spondyloarthropathy. According to Arthritis New Zealand, approximately 10% of members use emails therefore providing a total target sample of 400 participants who subsequently received an invitation to participate [23]. A response rate of 30-50% (120–200 participants) was anticipated [24],[25]. Ethical approval was obtained from Auckland University of Technology Ethics Committee (AUTEC). The survey was anonymous and self-administered.

### Survey development

The survey was developed and subject to pilot testing by all co-authors to ensure the relevance of the questions, and the final questionnaire was amended according to feedback. Three iterative revisions were conducted by the research team and these were based upon previous research [20], clinical experience, and current foot care recommendations [13]––[15]. The survey which was comprised of 18 questions was pilot-tested on five people with inflammatory arthritis and all co-authors. All co-authors agreed on the final version. Questions 1–5 were to elicit demographic information that included age, gender, New Zealand region of residence, ethnicity and current work status. Questions 6–8 sought to obtain information relating to current inflammatory arthritic condition, disease duration and current foot pain. Question 9 sought to elicit participant reports of the most important features to them when choosing footwear and the presented response options were based upon previous studies [17],[18],[26]. Questions 10 and 11 were designed to elicit information related to participants’ current footwear style most frequently worn during winter and summer months. A list of 14 styles of footwear was provided [27], with the addition of barefoot and socks. Participants were asked to rate how often they wear each footwear type (Never, Sometimes, Mostly, Always). Questions 12–16 relate to the role of healthcare professionals in providing footwear and foot orthoses. Question 17 was designed according to statements obtained from a previous study of people with rheumatoid arthritis in order to determine respondents’ previous experiences of footwear [20]. Participants had the opportunity at the end of the survey to make open-ended comments regarding their experience of footwear in relation to their arthritic condition (Question 18).

The survey utilised the online software, Survey Monkey® http://www.surveymonkey.com. This software allows users to self-create surveys and is easy to use with a large set of features [25]. Online surveys have the advantages of time-efficiency, reduced cost, automated data collection and an ability to overcome distance barriers in participant data collection [28],[29]. A hyperlink to the survey was placed on the Arthritis New Zealand website. The survey was open for approximately ten weeks between December 2013 and February 2014.

### Data analysis

Data were analysed using Statistical Package for Social Sciences (SPSS) V22.0 (IBM Corp., New York, USA). The primary analysis was descriptive statistics summarising survey results. Data from Survey Monkey® were manually entered into SPSS by one researcher (GB).

## Results

One-hundred and ninety-seven people responded to the survey. Twelve respondents were removed as they only completed questions related to demographic information.

### Demographics and clinical characteristics

The demographic and clinical characteristics are summarised in Table 1. The survey respondents were predominately women of European descent, aged between 46–65 years old, from the North Island of New Zealand. The majority reported having RA (n = 65, 35%) and/or OA (n = 106, 57%), and 68% (n = 125) reported having established disease (>5 years duration). Nearly half (n = 88, 48%) were in employment, 40% were retired and 16% were unemployed or involved in voluntary work. Over two thirds (n = 125, 72%) of respondents did not receive regular podiatric care, although 67% (n = 116) had been previously prescribed with insoles or orthotics from a health professional. Further, 18% of participants had been issued with therapeutic footwear from a private organisation contracted by the New Zealand government to provide footwear and orthoses to high-risk patients. Of those provided with therapeutic footwear, nearly one third (n = 9, 29%) reported that they do not wear their prescribed footwear and 45% (n = 14) reported that they wear only them occasionally.

**Table 1:** Participant demographic and disease characteristics

	**Male**	**Female**	**Total**
Gender, no. (%)	29 (16)	156 (84)	185 (100)
Age groups, no. (%)			
16-25 years	1 (3)	5 (3)	6 (3)
26-35 years	4 (14)	9 (6)	13 (7)
36-45 years	4 (14)	13 (8)	17 (9)
45-65 years	6 (21)	73 (47)	79 (43)
66-75 years	9 (31)	39 (25)	48 (26)
Over 75 years	5 (17)	17 (11)	22 (12)
Ethnicity, no. (%)			
Maori	1 (3)	5 (3)	6 (3)
European	27 (93)	142 (91)	169 (91)
Pacific Islander	1 (3)	2 (1)	3 (2)
Asian	0 (0)	7 (4)	7 (4)
Geographic location, no. (%)			
North Island	21 (72)	105 (67)	126 (68)
South Island	8 (28)	51 (33)	59 (32)
Employment status, no. (%)			
Employed	12 (41)	76 (49)	88 (48)
Voluntary work	3 (10)	14 (9)	17 (9)
Unemployed	1 (3)	11 (7)	12 (7)
Retired	14 (48)	58 (37)	72 (39)
On a benefit	2 (7)	20 (13)	22 (12)
Arthritic condition, no. (%)			
Rheumatoid Arthritis	11 (38)	54 (35)	65 (35)
Gout	5 (17)	7 (4)	12 (6)
Systemic Sclerosis	0 (0)	3 (2)	3 (2)
Fibromyalgia	2 (7)	13 (8)	15 (8)
Lupus	0 (0)	9 (6)	9 (5)
Osteoarthritis	13 (45)	93 (60)	106 (57)
Juvenile Idiopathic Arthritis	0 (0)	2 (1)	2 (1)
Psoriatic Arthritis	2 (7)	13 (8)	15 (8)
Spondyloarthropathy	3 (10)	11 (7)	14 (7)
Other	2 (7)	15 (10)	17 (9)
Disease duration, no. (%)			
6 weeks to 6 months	1 (3)	2 (1)	3 (2)
6 months to 1 year	0 (0)	9 (6)	9 (5)
1-5 years	8 (28)	40 (26)	48 (26)
5-10 years	6 (21)	38 (24)	44 (24)
More than 10 years	14 (48)	67 (43)	81 (44)
Foot Pain VAS (0–10), mean (SD)	5.0 (2.8)	5.5 (2.4)	5.4 (2.4)
Insoles or orthotics prescribed by podiatrist or other healthcare professional, no. (%)*	15 (63)	101 (68)	116 (67)
Has footwear from Orthotics Centre, no. (%)*	8 (33)	23 (15)	31 (18)
Wears footwear from Orthotics Centre, no. (%)			
All the time	3 (38)	5 (22)	8 (26)
Occasionally	3 (38)	11 (48)	14 (45)
Never	2 (25)	7 (30)	9 (29)
Receives regular podiatry treatment, no. (%)*	6 (25)	42 (28)	48 (28)
Last visited a podiatrist, no. (%)*			
Less than 1 month	2 (8)	16 (11)	18 (10)
1-2 months	0 (0)	6 (4)	6 (3)
3-6 months	4 (17)	20 (13)	24 (14)
7-12 months	3 (13)	12 (8)	15 (9)
13-24 months	2 (8)	13 (9)	15 (9)
More than 2 years ago	4 (17)	42 (28)	46 (27)
Never	9 (37)	40 (27)	49 (28)

*n = 173.

### Seasonal footwear habits, features of importance and experience of footwear

Table 2 demonstrates the frequency of footwear types reported by respondents as having been worn during the winter and summer months. Walking and athletic shoes were reported as the most frequently worn footwear type regardless of the season. In the summer, 42% of participants reported wearing sandals most often and 22% reported going barefoot most often. In winter, 20% of participants reported wearing boots while 26% reported wearing slippers most often. Table 3 presents participant responses related to the features that may be of importance to them when choosing footwear. Comfort, fit and support were frequently reported as being very important factors when choosing footwear. Colour was reported as the least important footwear attribute. Heel height was reported as very important by 54% of participants but 11% reported heel height as unimportant. Table 4 summarises agreement with statements previously made by people with arthritis, regarding their footwear experiences. Participants (63%) reported that they had difficulties with shoes (statement 1). The majority of respondents (63%) agreed with getting hot feet in the summer (statement 2) and the need for a sandal which could take a supportive insole (73%) (statement 5). The depth (statement 3) and width (statement 4) of the shoe were of less concern to participants. Eighty-five participants (46%) provided open-ended comments in relation to their footwear experiences (Question 18). Comments related to difficulties in finding appropriate footwear, dissatisfaction with footwear service provision, high cost of footwear and satisfaction/relief with eventual receipt of appropriate footwear.

**Table 2:** Type of footwear worn by respondents*

		**Never**	**Sometimes**	**Mostly**	**Always**
Winter, no. (%)	Sandal	146 (82)	30 (17)	1 (0.6)	1 (0.6)
	Mule	166 (93)	10 (6)	2 (1)	-
	Jandals/Flip-Flops	146 (82)	27 (15)	3 (2)	2 (1)
	Walking shoe	38 (21)	49 (28)	64 (36)	27 (15)
	Athletic shoe	25 (14)	65 (37)	64 (36)	24 (14)
	Moccasin	149 (84)	23 (13)	6 (3)	-
	Oxford shoe	143 (80)	22 (12)	12 (7)	1 (0.6)
	Therapeutic shoe	150 (84)	13 (7)	8 (5)	7 (4)
	Boot	78 (44)	64 (36)	29 (16)	7 (4)
	Ugg Boot	147 (83)	21 (12)	5 (3)	5 (3)
	High Heel	159 (89)	17 (10)	2 (1)	-
	Court shoe	99 (56)	65 (37)	13 (7)	1 (0.6)
	Slipper	55 (31)	77 (43)	33 (19)	13 (7)
	Backless slipper	135 (76)	24 (14)	14 (8)	5 (3)
	Socks	91 (51)	66 (37)	16 (9)	5 (3)
	Barefoot	123 (69)	47 (26)	7 (4)	1 (0.6)
Summer, no. (%)	Sandal	34 (19)	67 (39)	62 (36)	10 (6)
	Mule	157 (91)	14 (8)	2 (1)	-
	Jandals/Flip-Flops	94 (54)	52 (30)	19 (11)	8 (5)
	Walking shoe	45 (26)	62 (36)	49 (28)	17 (10)
	Athletic shoe	25 (15)	71 (41)	60 (35)	17 (10)
	Moccasin	157 (91)	14 (8)	2 (1)	-
	Oxford shoe	151 (87)	15 (9)	6 (4)	1 (0.6)
	Therapeutic shoe	154 (89)	13 (8)	5 (3)	1 (0.6)
	Boot	139 (80)	27 (16)	6 (4)	1 (0.6)
	Ugg Boot	165 (95)	8 (5)	-	-
	High Heel	153 (88)	19 (11)	1 (0.6)	-
	Court shoe	97 (56)	67 (39)	7 (4)	2 (1)
	Slipper	86 (50)	69 (40)	15 (9)	3 (2)
	Backless slipper	129 (75)	33 (19)	10 (6)	1 (0.6)
	Socks	103 (60)	59 (34)	10 (6)	1 (0.6)
	Barefoot	59 (34)	77 (45)	32 (19)	5 (3)

*n = 178 responses for footwear worn in winter, n = 173 responses for footwear worn in summer.

**Table 3:** Features of importance when choosing footwear, no. (%)

	**Not important**	**Slightly important**	**Of importance**	**Very important**
Comfort	-	-	16 (9)	169 (91)
Style	15 (8)	49 (27)	89 (48)	32 (17)
Fit	-	-	21 (11)	164 (89)
Support	3 (2)	7 (4)	36 (19)	139 (75)
Cost	13 (7)	41 (22)	87 (47)	44 (24)
Weight	9 (5)	51 (28)	77 (42)	48 (26)
Colour	22 (12)	58 (31)	78 (42)	27 (15)
Material	11 (6)	47 (25)	85 (46)	42 (23)
Fastenings	16 (9)	43 (23)	73 (39)	53 (29)
Nonslip	8 (4)	30 (16)	54 (29)	93 (50)
Heel height	20 (11)	14 (8)	51 (28)	100 (54)
Ease to put on	6 (3)	24 (13)	60 (32)	95 (51)

**Table 4:** Statements made about footwear experiences, no. (%)

		**Strongly disagree**	**Disagree**	**Agree**	**Strongly agree**
1	“I do have a lot of trouble with shoes; I have to have shoes that are very, very soft.”	17 (10)	46 (27)	77 (46)	29 (17)
2	“In the summer I want my feet to breathe. It’s getting hot in the shoes, so I don’t wear them in the summer.”	15 (9)	48 (28)	85 (50)	22 (13)
3	“The biggest problem I had getting shoes was the depth in the shoes, they weren’t tall enough around the toe area to accommodate the claw toes.”	46 (27)	63 (37)	42 (24)	20 (12)
4	“I’ve had to go up 1 shoe size and I’m like a double to triple fitting in the shoes.”	40 (24)	61 (36)	52 (30)	17 (10)
5	“Someone should invent a sandal that will take the insole and support your foot. . . . You put up with your feet being roasted in the summer.”	15 (9)	31 (18)	70 (41)	55 (32)

## Discussion

The aim of the study was to determine the seasonal influences on footwear habits in people with inflammatory arthritis in New Zealand. Previous qualitative studies highlighted issues around seasonal footwear choices for people with RA-related foot problems [20],[22]. To our knowledge, the current findings are the first to report seasonal influences on the footwear worn by people with inflammatory arthritis.

We found that therapeutic footwear was reported as being worn by a small number of participants regardless of the season. Despite the benefits of therapeutic footwear [11],[16] this type of footwear was not widely worn by patients in the current study. Additionally there are known factors relating to poor use of therapeutic footwear and reasons why therapeutic footwear is deemed to be unacceptable [15],[20],[21]. Williams et al. [15] identified therapeutic footwear as being the only intervention that replaces something that is normally worn as an item of clothing and therefore reinforces the stigma of foot deformity and disability. In addition to body image issues, Hendry et al. [20] reported that patients feet become over-heated in closed in therapeutic footwear, and Otter et al. [21] reported that some patients discontinued using therapeutic footwear either because their foot symptoms had resolved or because they had foot surgery. Adherence to podiatric intervention strategies, in particular therapeutic footwear, appears to be an important consideration in inflammatory arthritis. There is good evidence to suggest that healthcare priorities and preferences of care provided are different for patients who have arthritis [30]. Awareness of these issues has the potential to improve patient care through shared clinical decision making [31],[32]. Issues with non-adherence to therapeutic footwear has previously been reported in studies from the USA [33], UK [21],[34], Turkey [35], Australia [36], The Netherlands and Spain [37].

In evaluating footwear worn in winter and summer, we found that athletic and walking shoes were the most popular style reported regardless of season. Athletic and walking shoes have been reported as the most comfortable option for people with RA [38], OA [39] and gout [10],[18]. A previous study reported that athletic shoes were an acceptable alternative to off-the-shelf therapeutic or orthopaedic footwear for people with RA-related forefoot pain [40], suggesting that healthcare professionals should consider walking and athletic shoes for routine footwear advice in people with inflammatory arthritic conditions.

The most striking seasonal difference was the wearing of sandals, which were reported as being worn frequently by 42% of respondents during the summer. Previous studies have reported similar findings [17],[41],[42], and may be in part due to forefoot structural deformities, severe bunions and clawing of the lesser toes often observed in inflammatory arthritic conditions. Sandals are defined as shoes with a sole that is fastened to the foot by thongs or straps [17]. Despite the availability of good quality sandals, nearly three-quarters of respondents agreed with the statement “Someone should invent a sandal that will take the insole and support your foot…”. This may indicate a lack of awareness of the more supportive, higher quality sandals that are commercially available. It is also possible that the cost of high quality supportive or orthotic-friendly sandals is a prohibitive factor for many people with arthritic conditions [17],[18],[20].

In the current study, participants reported that comfort, fit and support were reported as the most important features when choosing footwear, and the majority of participants agreed with statements regarding the need for a softer and more supportive shoe. Previous studies in people with RA [17],[26] and gout [18] also reported that comfort, fit and support were important, which may suggest that people with inflammatory arthritis prioritise these features due to disease-related foot problems. The majority of respondents also agreed with statements regarding the feet overheating in closed-in footwear during the summer. The provision of publically funded therapeutic sandals and education regarding the availability of high quality, supportive, commercially available sandals, may be warranted.

The study has limitations. The study was undertaken in New Zealand and may not reflect footwear trends in other developed countries. The survey was only available electronically thus excluding those without access to a computer or internet connection. Therefore the results may not be representative of the wider population with inflammatory arthritis in New Zealand. The elicitation of self-reported responses does not necessarily represent ‘actual’ footwear habits/experiences, but rather respondents’ opinions/perceptions of their habits at the time of survey completion. Given the reported popularity of wearing athletic shoes, walking shoes and sandals in people with inflammatory arthritis, further research investigating the long term effects of commercially available footwear on foot pain, impairment and disability may be warranted.

## Conclusions

The current findings are the first to report seasonal influences on the footwear habits of people with inflammatory arthritic conditions. Therapeutic footwear was reported as being worn by a small number of participants regardless of the season and may reflect issues with adherence, body image, fit and comfort. Athletic and walking shoes were the most popular style regardless of season and may reflect people with inflammatory arthritic conditions choice of footwear. The wearing of sandals was popular during the summer that suggests people with inflammatory arthritis wear sandals in order to better accommodate forefoot deformity and associated pain, impairment and disability. The popularity of sandals may also be due to feet getting hot in closed-in footwear and education is needed regarding the availability of high quality orthotic-friendly sandals. Healthcare professionals should be aware of seasonal variations in footwear worn by people with inflammatory arthritis and assist their patients in accessing appropriate footwear to reduce non-adherence.

## Competing interests

The authors declare that they have no competing interests.

## Authors’ contributions

KR and GH conceived the study protocol. KR, GH and GB designed the survey. GB piloted and finalised the survey and inputted the data. ABR conducted the statistical analysis and ABR, KR and GH interpreted the findings. ABR, KR and GH drafted the manuscript with input from GB and the final version was read and approved by all co-authors.
